# On the importance of being clear about the level of analysis of interest: An illustration using the case of self‐compassion

**DOI:** 10.1111/jopy.12924

**Published:** 2024-03-10

**Authors:** Anabel Büchner, Christina Ewert, Cosma F. A. Hoffmann, Michela Schröder‐Abé, Kai T. Horstmann

**Affiliations:** ^1^ Institute of Psychology Humboldt‐Universität zu Berlin Berlin Germany; ^2^ Department of Psychology University of Potsdam Potsdam Germany; ^3^ Institute of Psychology University of Greifswald Greifswald Germany; ^4^ Department of Psychology University of Siegen Siegen Germany

**Keywords:** ergodicity, experience‐sampling, self‐compassion, states, traits, within‐person analysis

## Abstract

**Objective:**

Theories about within‐person (WP) variation are often tested using between‐person (BP) research, despite the well‐established fact that results may not generalize across levels of analysis. One possible explanation is vague theories that do not specify which level of analysis is of interest. We illustrate such a case using the construct of self‐compassion. The factor structure at the BP level has been highly debated, although the theory is actually concerned with relationships at the WP level.

**Method:**

Multilevel confirmatory factor analysis was applied to experience‐sampling data of self‐compassion (*N* = 213, with *n* = 4052 measurement occasions).

**Results:**

At both levels of analysis, evidence for a two‐factor model was found. However, the factors were moderately related at the WP level (*r* = 0.37, *p* < 0.001) but largely independent at the BP level (*r* = 0.04, *p* = 0.696). Exploratory analyses revealed considerable heterogeneity in the WP relationship among individuals.

**Conclusion:**

We discuss how our results provide new impulses to move the debate around self‐compassion forward. Lastly, we outline how the WP level—which is of major interest for self‐compassion and other constructs in psychology—can guide the conceptualization and assessment to promote advancements of the theory and resulting applications.

## 1 INTRODUCTION

Much research in psychology is concerned with the study of interindividual (i.e., between‐person) differences. This focus is rooted in the history of psychological research. In the first half of the 20th century, between‐person (BP) differences were of most interest in selection decisions in the military or educational settings (see Hamaker, 2012 for a historical overview). Likewise, classical test theory by Lord and Novick (1968)—on which most scales to measure psychological constructs are based—defines true and error terms with respect to inter‐ instead of intra‐individual (i.e., within‐person) variation, as pointed out by Molenaar (2004). At the same time, many psychological theories and their applications (e.g., interventions, psychotherapy) are concerned with within‐person (WP) variation. For example, interventions build on the assumption that a change in one variable (e.g., self‐esteem) will lead to change in another variable (e.g., depressive symptoms).

It is important to distinguish between associations at the BP and WP levels as results do not necessarily hold across levels of analysis (Borsboom et al., 2003; Fisher et al., 2018; Molenaar, 2004). In fact, results can only be transferred from one level of analysis to another under very specific conditions, which are seldomly, if ever, met in psychological research (Hamaker, 2012; Molenaar, 2004). Yet, as others have noticed (e.g., Beal & Gabriel, 2019; Fried, 2020), it seems that results obtained through the study of BP differences are still often interpreted as evidence for theories that are primarily concerned with WP processes. This results in the fact that empirical findings do not speak to the theories that they are supposed to test, thereby providing no or even the wrong kind of evidence for the tested theories.

In this paper, we underline the need for psychological theories to clearly state at which level of analysis the effect of interest is located. We use the construct of self‐compassion to illustrate a case in which a theory's lack of differentiation between the WP and BP levels may have contributed to most research being conducted at a suboptimal level of analysis, and to ambiguity about which empirical evidence contributes meaningfully to the understanding of the theory under investigation. Self‐compassion refers to a caring attitude towards the self in difficult times (Neff, 2003a, 2003b) and has received great attention in psychological research. Although self‐compassion appears to be a promising construct for interventions (e.g., Gilbert, 2009; Neff & Germer, 2013; Kirby et al., 2017), its measurement and conceptualization are highly debated. Despite numerous studies being published on this issue, there is still disagreement about the computation and interpretation of the scores of the most used scale to assess self‐compassion (e.g., Muris & Otgaar, 2020, 2022; Neff, 2020, 2022). The resulting divergent procedures hamper scientific progress in the area. We suggest focusing on the level of analysis might provide a key to moving the debate forward: While most studies focus on the structure of self‐compassion at the BP level, the theory of self‐compassion is actually concerned with associations at the WP level. To empirically demonstrate the importance of distinguishing both levels, we investigated the WP and BP structure of self‐compassion as a state in daily life. Moreover, we outline how the WP level—which is of major interest for self‐compassion and many other constructs in psychology—can guide the conceptualization and assessment of constructs to promote advancements of the theory and resulting applications.

### 1.1 Differentiating between traits and states

To study BP differences, psychological constructs are routinely measured as stable traits. Most commonly, traits are assessed with global self‐reports which ask individuals about their typical thoughts, feelings, and behaviors (e.g., Costa & McCrae, 1992). Since traits are assumed to be relatively stable across time, they only need to be assessed once per study. Due to their economical assessment and ability to predict important outcomes (e.g., Roberts et al., 2007), traits are popular in psychological research and applications. Nevertheless, it is widely acknowledged to date that cross‐sectional trait measures provide only limited knowledge about WP processes (Borsboom et al., 2003; Fisher et al., 2018; Molenaar, 2004). Yet, WP processes are often of interest, for example, to gather essential information for interventions.

To study WP processes, psychological constructs are often measured as states. Since states are assumed to vary within persons across time and situations, they are commonly assessed in intensive longitudinal studies by asking people about their momentary thoughts, feelings, and behaviors (Fleeson, 2001; Horstmann & Ziegler, 2020). The recent rise of studies on WP processes was not only fueled by growing awareness that results can differ across levels of analysis but also by technological and methodological advances. Most influential was arguably the introduction of the experience‐sampling method (ESM; Larson & Csikszentmihalyi, 1983), which consists of the repeated measures of a set of variables in daily life, and its convenient implementation through the widespread use of smartphones.

Although state measures should, by definition, capture substantially more WP variance than trait measures, WP variance can only be statistically disentangled from BP variance in repeated assessments. In repeated observations of states, the individual mean over time (i.e., aggregated state score per person) captures BP differences and has been suggested to represent an accurate estimate of one's trait level (Fleeson, 2001). The momentary deviations of the aggregated state score then represent the variable, state‐like part, which is suited to study WP associations. In contrast, responses to cross‐sectional questionnaires contain a blend of BP and WP variation with the specific ratio being determined by the stability of the construct under investigation (Cattell, 1978).

### 1.2 Better methods alone may not be enough

Given the technological and methodological advances in the last years, why does it seem that theories about WP processes are still commonly tested using BP research? One possible explanation may be a lack of clarity about the level of analysis of interest in psychological theories. The aim of psychological theories is to explain, predict, or control observable phenomena such as the relationships between variables or differences between individuals. Psychological theories have been criticized as being too vague in the sense that they, for example, do not spell out the precise conditions under which hypothesized effects are expected to occur (Fried, 2020). This also includes a common absence of specifying at which level of analysis the effect is located.

The fuzziness of theories is also reflected in insufficiently defined concepts that represent the basic unit of theories (Bringmann et al., 2022; Flake & Fried, 2020; Scheel et al., 2021). Concepts refer to all kinds of ideas, categories, or elements that are grouped together based on shared properties and help understanding the phenomena of interest. Psychological constructs such as personality traits represent a special type of concepts that cannot be directly observed but have to be inferred from empirical data. For psychological constructs, the lack of clarity about the level of analysis of interest may be expressed in the fact that almost all constructs in personality psychology are conceptualized and measured as traits even if the corresponding theories are concerned about WP variation. For example, this mismatch between theory and operationalization has been pointed out for constructs such as loneliness (Buecker et al., 2023) and narcissism (Edershile & Wright, 2021). In this paper, we use the construct of self‐compassion to underline the need for psychological theories to enhance clarity about the level of analysis and to conceptualize and assess the constructs in alignment with the level of analysis of interest.

### 1.3 The construct of self‐compassion

In the early 2000s, self‐compassion was initially conceptualized in psychological research as a protective trait (Neff, 2003a, 2003b). According to Neff's definition (Neff, 2003a, 2003b), self‐compassion consists of three bipolar components^1^ that each have a positive and a negative pole. The first component describes encountering oneself in difficult times with acceptance, sympathy, and care (*self‐kindness*) vs. harshly criticizing oneself in such moments (*self‐judgment*). The second component involves remembering that all humans are imperfect and go through difficult times (*common humanity*) vs. experiencing one's suffering as unique such that feelings of separation from others arise (*isolation*). The last component refers to holding negative emotional states in balanced awareness (*mindfulness*) vs. being so overwhelmed by unpleasant emotions that one loses the broader perspective (*overidentification*). According to the initial theory, the components depend on each other and jointly form the construct of self‐compassion.

In recent years, the construct has attracted great interest in psychological research as indicated by an exponentially growing number of citations of articles on self‐compassion (Muris & Otgaar, 2020). Meta‐analyses support the protective nature of self‐compassion by linking it to well‐being (Zessin et al., 2015), mental health (MacBeth & Gumley, 2012; Marsh et al., 2018; Turk & Waller, 2020), adaptive coping (Ewert et al., 2021), physical health and health behavior (Phillips & Hine, 2021). Importantly, self‐compassion is considered a “skill that can be learned” (Germer & Neff, 2013, p. 856), and its malleable nature is empirically supported by studies reporting that self‐compassion can be enhanced relatively quickly through practice (Ferrari et al., 2019; Kirby et al., 2017). As such, the construct became a popular target in interventions (Neff et al., 2020; Neff & Germer, 2013) and psychotherapy (Gilbert, 2009; Wilson et al., 2019).

### 1.4 The debate around self‐compassion

However, self‐compassion, and precisely its measurement with the Self‐Compassion‐Scale (SCS; Neff, 2003b), has been debated for years. The discussion started with the factor structure of the SCS (for an overview, see Muris & Otgaar, 2020; Neff, 2020). During the scale construction, it turned out that the positive (self‐kindness, common humanity, and mindfulness) and negative (self‐judgment, isolation, and overidentification) poles of the components seemed to be no opposites of each other but instead were best represented by an individual factor each (Neff, 2003b). Moreover, the general factor that should represent the construct of self‐compassion did not replicate across studies (e.g., Hupfeld & Ruffieux, 2011; Williams et al., 2014). Simultaneously, several studies suggested that the positive and negative components form two separate factors (e.g., Coroiu et al., 2018; Costa et al., 2016; López et al., 2015), referred to as compassionate self‐responding (CS) and uncompassionate self‐responding (UCS) in the literature. Results suggesting that the SCS captures two distinct constructs—CS and UCS—build the foundation for the debate that centers around two questions.

The first question concerns how the scale scores of the SCS should be computed. Factor solutions with two separate factors for CS and UCS suggest two total scores, whereas most studies still use a single score for the SCS (Muris & Otgaar, 2022). This likely stems from the defense of the use of the total score over the last years (e.g., Neff, 2016; Neff et al., 2017, 2019; Tóth‐Király et al., 2017). Nevertheless, a growing body of papers uses two scores instead of a single total score (e.g., Lian et al., 2023; Pandey et al., 2019).

The second question concerns the role of the UCS components for self‐compassion. Some argue that the USC items should be excluded from the scale because they do not measure self‐compassion but psychopathology or neurotic tendencies (Geiger et al., 2018; Muris, 2016; Muris et al., 2019; Muris & Petrocchi, 2017; Pfattheicher et al., 2017). This would constitute a jangle fallacy (i.e., different labels being used for the same construct) and predictor‐criterion contamination when the relationship between self‐compassion and psychopathology is examined. In contrast, others argue that USC is distinct from neuroticism or psychopathology (Neff, 2020; Neff et al., 2018) and represents an integral part of “self‐compassion as a holistic state of being” (Neff et al., 2019; p. 29). The ongoing debate diminishes comparability across studies because scale scores are computed and interpreted inconsistently.

### 1.5 How to move forward in the debate around self‐compassion?

As Ferrari et al. (2022) already suggested, this debate may not be resolved by yet another study providing empirical evidence for or against one of the positions. Instead, we highlight a critical but so far mostly overlooked aspect that may have the potential to move the debate in a fruitful direction: The debate is based on research on self‐compassion measured as a trait, and thus focuses mostly on the factor structure at the BP level; however, the relationships between the components are assumed to unfold at the WP level. That is, according to the theory, the components depend on and enhance each other within an individual at the very moment. For example, mindfulness is considered an essential condition for the experience of self‐compassion, as Neff stated “[…] a certain degree of mindfulness is needed in order to allow enough mental distance from one's negative experiences that feelings of self‐kindness and common humanity *can arise* [emphasis added]” (Neff, 2003b, p. 89). Since feelings always arise within individuals, this quote illustrates that the relations between the components must be located at the WP level.

Yet, it has not yet been spelled out explicitly that the WP level is of major interest for testing the theoretically assumed interrelations of the construct. Instead, there is a lack of clarity about the level of analysis of self‐compassion, which may have caused confusion in the past. For example, it was argued that one of the best ways to determine whether CS and UCS operate jointly or independently is by investigating self‐compassion as a state (Neff, 2020, p. 1906). If measured cross‐sectionally as a state, the high correlation between US and UCS could indeed suggest that they capture the same construct (*r*s >0.83 in 2 samples, Neff et al., 2021), which is not necessarily implied by the magnitude of the correlations based on cross‐sectional trait measures (e.g., *r* = 0.08 to *r* = 0.79 in 11 samples; Halamová et al., 2021). While Neff (2020) argued that those findings contribute to the validity of the total score of trait self‐compassion, Ferrari et al. (2022) suggested that the stronger relationship between CS and UCS in state compared to trait reports implies that the time frame matters for the strength of the relationship. Yet, since state and trait measures should differ in the amount of WP vs. BP variance they capture, we believe that those findings indicate differences across levels of analysis.

## 2 THE PRESENT RESEARCH

To investigate whether the structure of self‐compassion meaningfully differs across levels of analysis, we applied multilevel confirmatory factor analysis (MCFA) to ESM data in which self‐compassion was measured as a state in daily life. In a preregistered analysis, we separately compared competing factor models reported in the literature on trait self‐compassion at the WP and BP levels. However, while the WP models in the MCFA capture the average WP structure across individuals, research on effect (Brose et al., 2015) or the Big Five dimensions (Beck & Jackson, 2020) highlights that individuals tend to substantially vary in their WP structure. Importantly, understanding these variations can pave the way for theoretical advancements. To illustrate this potential for the construct of self‐compassion, we conducted exploratory analyses on interindividual differences in the intraindividual relationship at the manifest level. Specifically, we investigated how strongly individuals varied in the WP relationship between CS and UCS, and how the composite scores of CS and UCS may be associated with interindividual differences therein.

## 3 METHOD

The present research used data that were collected in 2018 as part of the project “MindOn.” The same dataset was used in other manuscripts (Ewert et al., 2022) but the analyses carried out in the present research have not been reported before. The analyses were preregistered on the Open Science Framework (OSF) (https://osf.io/2p7sq), and all deviations from the preregistration are made explicit. The data, R code, supplementary material, and an overview of all assessed variables are also available via the OSF (https://osf.io/fbcwp/).

### 3.1 Procedure

The data collection consisted of three parts. First, participants came to the laboratory to complete several trait measures, which were not analyzed in the current study. Second, participants completed an ESM part on their smartphone or tablet via the app movisens (movisens GmbH, see www.movisens.com). The ESM part started the following day and consisted of three surveys a day for 7 days. The surveys were scheduled at random within three‐time blocks à 4 h between 10 am and 10 pm with at least 1 h between prompts. If participants did not respond immediately, they received two reminders within an interval of 10 min each. Surveys that had been started were automatically closed after 15 min. Finally, participants were invited via email to fill out the initial survey with the trait measures once more. The study compensation was tied to response rate (>70% or >90%), offering either course credits or financial compensation (max. 20 euros).

### 3.2 Sample

The final sample consisted of 213 participants with a total of 4052 measurement occasions. As preregistered, we excluded measurement occasions with missing data in the self‐compassion items (*n* = 9) and participants with less than three measurement occasions (*n* = 3). In addition to these preregistered criteria, measurement occasions with less than 1 h distance to the prior prompt were excluded (*n* = 86) as in the study set up 1 h was defined as the minimal time lag between two prompts, and deviations from this must have been caused by technical malfunctioning. On average, each participant provided 19.02 reports in the final sample (*SD* = 2.44, *Range* [3; 21]), leading to a response rate of 90.59%. Most of the participants identified as female (86%) and were students (94.79%). The average age of the sample was 23.32 years (*SD* = 0.39, *Range* [16; 59]).^2^

### 3.3 State measure of self‐compassion

Self‐compassion was measured with 12 items included in the short form of the Self‐Compassion‐Scale (SF‐SCS; Raes et al., 2011). For the current study, the items were adapted from the German version (Hupfeld & Ruffieux, 2011) of the SCS (Neff, 2003b) so that they referred to self‐compassion in the current moment. That is, the part of the original item text that described the instance of suffering (e.g., “When things are going badly for me…”; Neff, 2003b) was left out, and instead, present moment language was included (e.g., “I'm feeling…”). We note that this adaptation may have changed the assessed construct, a concern that we address later in the paper (in the section “robustness checks”). Participants were instructed to rate the extent to which the items applied to them in the current moment on a 5‐point rating scale (1 = not at all to 5 = strongly). A sample item for each subscale is given in Table 1. The descriptive statistics of the items can be found in the OSF (Table S3).

**TABLE 1:** Example items for each subscale as used in the current study.

Subscale	Adapted example item of the SF‐SCS
Self‐kindness (SK)	I'm trying to be understanding and patient towards those parts of my personality that I do not like
Mindfulness (MI)	I'm trying to keep my emotions in balance
Common humanity (CH)	I'm trying to see my flaws as part of the human condition
Self‐judgment (SJ)	I'm disapproving and judgemental about my own flaws and inadequacies
Overidentification (OI)	I'm obsessing and fixating on everything that is wrong
Isolation (IS)	I'm feeling like most other people are probably happier than I am

*Note*: Each subscale was assessed with two items. The items were rated on a scale from 1 (*not at all*) to 5 (*strongly*).

### 3.4 Data preparation and analysis

Data were prepared and analyzed in R 4.3.2 (R Core Team, 2023) with tidyverse 1.3.1 (Wickham et al., 2019), lavaan 06.‐17 (Rosseel, 2012), and lme4 1.1.27.1 (Bates et al., 2015).

#### 3.4.1 Data preprocessing and preliminary analyses

The items of the UCS scales (self‐judgment, isolation, and overidentification) were reverse scored. To estimate the ICC, random intercept models were specified for each item. The ICC was calculated by dividing the BP variance by the total variance.

#### 3.4.2 MCFA

The two‐level CFA accounts for the nesting of the measurement occasions (level 1) in the participants (level 2) through the inclusion of random intercepts. At level 2 (i.e., BP level), stable differences between participants (i.e., the latent person‐mean across all measurement occasions) were modeled. At level 1 (i.e., WP level), the deviations from the latent person‐mean across occasions were modeled, meaning data from all measurement occasions from which stable differences between participants were removed. The MCFA with random intercepts represents the *average* WP structure since only the intercepts (i.e., the person‐means) are allowed to vary across participants.

#### 3.4.3 Model specification and estimation

Latent variables were identified by fixing the loading of the first indicator to 1. The loadings of the measurement models for the subscales were also fixed to 1 as those models were only composed of two items each and, hence, they would not be independently identified otherwise. We decided to include the constraints on the loadings and not on the variance because we were interested in whether all latent factors would possess significant variance. To account for non‐normality in the data, the models were estimated using robust maximum likelihood (MLR) estimation.

#### 3.4.4 Model evaluation

The competing models were separately evaluated at the BP and the WP levels by setting up partially saturated models (Ryu & West, 2009). As pointed out by Ryu and West (2009), a separate evaluation of models at both levels in an MCFA is important if the sample size differs considerably between levels because otherwise the misfit at the level with the lower sample size would be masked by the model specified at the level with the greater sample size. A saturated model includes the maximum number of possible parameters, meaning that it perfectly reproduces the variances and covariances in the dataset. Since it fits the data perfectly, specifying a saturated model at the level of analysis that is currently not being evaluated means that this level does not impact the joint model fit. In partially saturated models, a saturated model is specified at level 1 when the aim is to evaluate a model at level 2, and vice versa. Through this approach, the fit at the specific level of interest can be isolated and assessed without interference from the fit at the other level. To evaluate the goodness of the fit of the partially saturated models, we followed Ryu and West (2009) and calculated the level‐specific CFI and the level‐specific RMSEA. Moreover, we also followed Ryu and West's (2009) decision to focus on the CFI and the RMSEA because both fit indices demonstrated particularly good performance (West et al., 2012).^3^

To select the best‐fitting model, we preregistered up to four consecutive decision criteria. For example, only models that met criterion 1 would be evaluated in terms of criterion 2, and only if more than one model would meet criterion 1. Our minimal criterion (i.e., criterion 1) was defined as an acceptable model fit in terms of standard cut‐offs for single‐level factor analysis (CFI >0.90, RMSEA <0.08, Hu & Bentler, 1999). Additionally, all latent variables (except residual variances) were required to demonstrate significant variance (*p* < 0.05). Criterion 2 stated that models with one or two general factors will be chosen over the model with six correlated factors. The subsequent criteria did not become relevant and are hence not presented here but can be found in the pre‐registration.

#### 3.4.5 Exploratory model modification

Because none of the confirmatory models provided a satisfying fit without modifications, we followed with the exploratory analysis part of our preregistration, which consisted of testing models with two general factors stepwise (i.e., first the measurement model and then the structural model) and the tweaking of models with a close fit (i.e., including correlated residuals) to investigate and model the misfit.

#### 3.4.6 Correspondence of both levels

We evaluated the configural correspondence in terms of whether the same model(s) provided an acceptable fit at both levels. We evaluated the structural correspondence in terms of whether there seemed to be no substantial descriptive difference in the correlation between the factors at both levels.

#### 3.4.7 Deviations from the preregistration

We did not conduct the preregistered invariance tests as it would have been unreasonable to constrain the model parameters to be equal across the WP and BP levels due to the large difference in sample sizes of both levels (Ryu & West, 2009). For a more detailed explanation, see the OSF.

### 3.5 Competing models of self‐compassion

**FIGURE 1:**
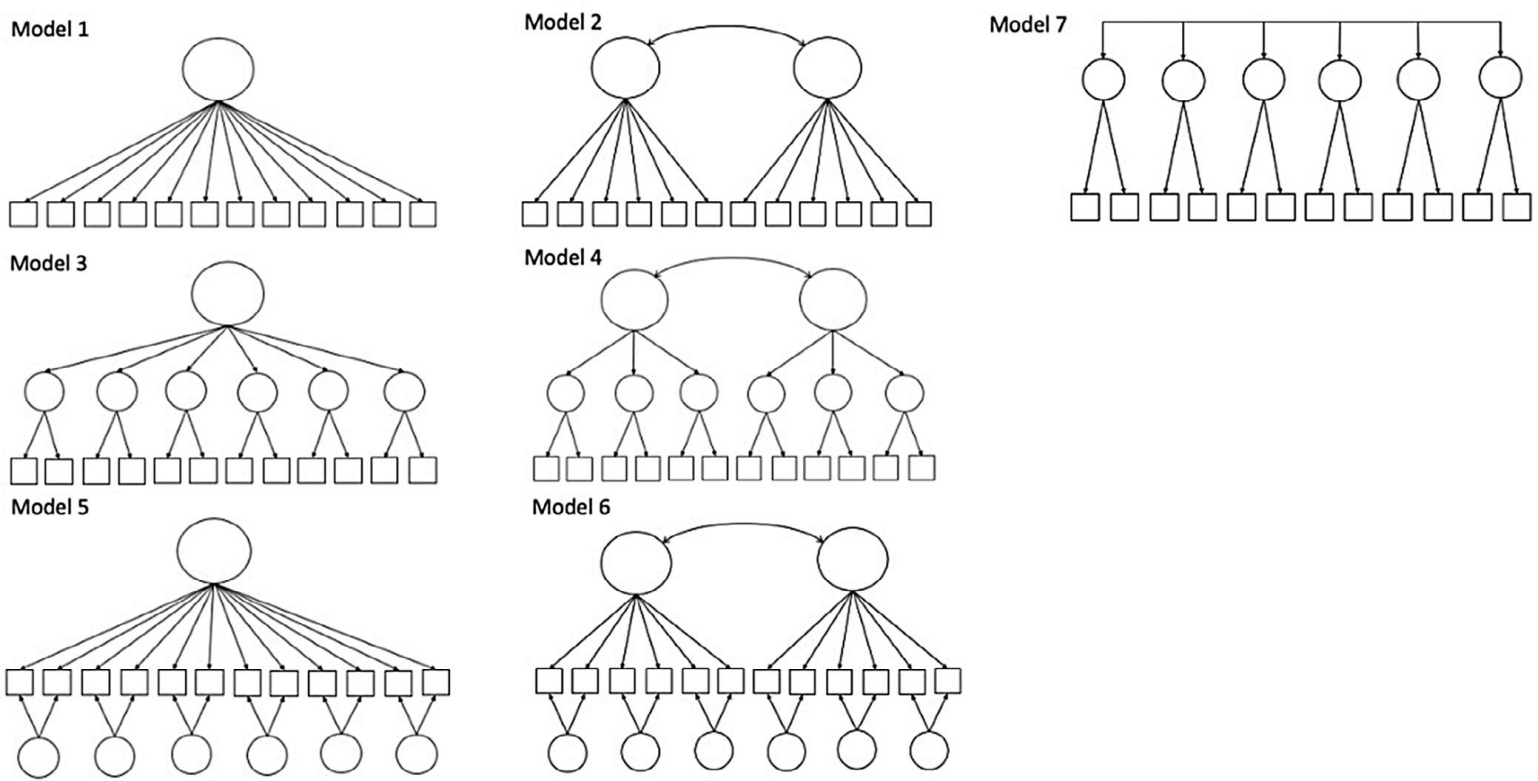
Schematic depiction of the seven competing models of self‐compassion.

Based on past studies on trait self‐compassion, seven models were tested against one another (cf. Figure 1). Overall, the competing models vary in (a) whether they include specific factors for the subscales or not, (b) the number of general factors, and (c) whether specific and general factor(s) (if included), were arranged as higher‐order or bifactor model. A more detailed description of the models and their corresponding support by past studies is given below.

Model 1 is a simple one‐factor model that only includes a general self‐compassion factor. This model has received no support from past studies. We still tested Model 1 because it is implicitly assumed whenever a total score is estimated by averaging across all items, which is mostly done for the SCS as well as the SF‐SCS (Muris & Otgaar, 2020).

The corresponding two‐factor solution is Model 2, in which the positive and negative items form two separate but correlated factors. The two‐factor model has been reported as the best‐fitting model in some studies on the SCS (Coroiu et al., 2018; Costa et al., 2016; Kumlander et al., 2018; López et al., 2015) and the SF‐SCS (Babenko & Guo, 2019; Hayes et al., 2016).

Model 3 is a higher‐order model, in which the intercorrelations of the six factors are explained by an overarching self‐compassion factor. This model was initially introduced for the SCS (Neff, 2003b) and for the SF‐SCS (Raes et al., 2011), but has not received conclusive support.

Model 4 is the corresponding two‐factor solution, that is, a higher‐order model with two‐correlated factors. In Pfattheicher et al. (2017), Model 4 was the only model with at least one general factor that provided an acceptable fit for the SCS.

Model 5 is a bifactor model in which all items load on the general self‐compassion factor and on the specific factor of their subscale. While a few studies found support for the bifactor model for the SCS (Cleare et al., 2018; Neff et al., 2017), others did not (e.g., Brenner et al., 2017; Coroiu et al., 2018; Kumlander et al., 2018). To our knowledge, no study has yet investigated the fit of a bifactor model for the SF‐SCS.

Model 6 is a bifactor model with two‐correlated general factors, also known as the two‐tier model. In several samples, the two‐factor solution of the bifactor model provided a better fit for the SCS than the corresponding one‐factor solution (Brenner et al., 2017; Halamová et al., 2021).

Model 7 consists of six correlated factors and is the best‐supported model in the literature on self‐compassion. The six‐factor model provided an acceptable fit in most studies that investigated the factor structure of the SCS and the SF‐SCS. However, because it is the least parsimonious model, models with one or two general factors are usually preferred. Yet the six‐factor model is also frequently the only model that provides an acceptable fit, and this holds for the SCS (Arimitsu, 2014; Azizi et al., 2013; Garcia‐Campayo et al., 2014; Hupfeld & Ruffieux, 2011; Lee & Lee, 2010; Petrocchi et al., 2014; Wiliams et al., 2014) and the SF‐SCS (Garcia‐Campayo et al., 2014; Uršič et al., 2019).^4^

## 4 RESULTS

The descriptive statistics (Table S3) and items correlations (Table S4 and Figure S1) can be found in the OSF.

### 4.1 Between‐person models

Table 2 presents the model fits of the competing models at the BP level. Only the model with six correlated factors (Model 7) fulfilled the minimum criterion for an acceptable model fit and significant factor variances. However, this model was rendered invalid due to an ultra‐Heywood case, with a correlation of *r* = 1.03 between the subscales of self‐judgment and overidentification (cf. Table S4 in the OSF). To address this issue, we tested an exploratory, modified version of Model 7 in which the items of self‐judgment and overidentification loaded onto the same factor. The five‐factor model fitted equally well as the six‐factor model but showed no anomalies and was therefore preferred. The correlations of the five factors at the BP level are displayed in Table 3 (below the diagonal). The factors of CS correlated highly with each other (*r* = 0.79 to *r* = 0.83) as did the factors of UCS (*r* = 0.80). However, most correlations between the factors of CS and UCS were non‐significant, and some were even small negative. Given that the UCS items were reverse scored, only positive correlations between the factors would be predicted by the theory of self‐compassion. Thus, these correlations do not support a general self‐compassion factor but suggest separate factors for CS and UCS.^5^

**TABLE 2:** Model fits of the competing models at the BP levels.

No.	Model type	CFI	RMSEA	df	*χ* ^*2*^	*p*	Note
1	One‐factor model	0.508	0.064	54	887.139	<0.001	–
2	Two‐correlated factor model	0.815	0.039	53	371.513	<0.001	–
2^a^	Two‐correlated factor model with three residual correlations	0.910	0.028	50	207.284	<0.001	Exploratory model
3	One higher‐order factor model	0.753	0.045	54	476.741	<0.001	SJ n.s. and OI negative variance
4	Two‐correlated higher‐order factor model	0.887	0.031	53	251.315	<0.001	SK, CH, SJ n.s. and OI negative variance
5	One bifactor model	0.785	0.045	48	409.836	<0.001	SJ n.s. and OI negative variance
6	Two‐correlated bifactor model	0.917	0.028	47	197.225	<0.001	SK, SJ n.s. and OI negative variance
7	Six‐correlated factor model	0.904	0.031	45	208.217	<0.001	SJ and OI correlate >1.0
7^a^	Five‐correlated factor model	0.903	0.029	51	210.260	<0.001	Exploratory model

*Note*: Robust values for the model fits are displayed. n.s. = not significant at *p* < 0.05.

Abbreviations: OI, overidentification; SJ, self‐judgment; SK, self‐kindness.

^a^ Modified version of the preregistered model.

**TABLE 3:** Correlations of the five factors at the BP and WP levels.

	1	2	3	4	5
1 Self‐kindness (SK)		0.50	0.81	0.52	0.46
2 Mindfulness (MI)	0.79		0.87	*−0.02*	*0.03*
3 Common humanity (CH)	0.83	0.79		*0.05*	*0.08*
4 Self‐judgment and overidentification (SJOI)	*0.14*	*−0.13*	−0.19		0.81
5 Isolation (IS)	0.22	*0.02*	*−0.07*	0.80	0.82

*Note*: Correlations at the BP level are displayed below the diagonal and correlations at the WP level above the diagonal. At the WP level, the covariance matrix of the model was not positive definite. However, the correlations of the four‐factor model in which MI and CH were combined suggest that this issue did not substantially impact the estimations of the factor correlations (cf. Table S6 in the supplementary material). Non‐significant (*p* > 0.05) correlations are in italics.

Models with two general factors consistently fitted better than models with one general factor. Among models with at least one general factor, only the two bifactor models demonstrated an acceptable fit (Model 6). However, Model 6 did not meet criterion 1 because three specific factors had either non‐significant or negative variances.^6^ This suggests that some of the specific factors did not explain substantial variance in the items above the general factors, suggesting that a simple two‐factor model may be better suited to capture the covariance structure in the data.

As the two‐factor model (Model 2) had a close fit, we followed the preregistered exploratory steps to investigate the source of the misfit. The separate investigation of each measurement model revealed that neither only the CS items (*χ* ^*2*^ = 129.57, *p* < 0.001, CFI = 0.811, RMSEA = 0.062) nor the UCS items (*χ* ^*2*^ = 97.582 *p* < 0.001, CFI = 0.875, RMSEA = 0.059) provided an acceptable fit. Following the modification indices, three residual correlations were added to reach an acceptable model fit. Specifically, a residual correlation between the items of the subscales of isolation and mindfulness were included as well as between item 1 (self‐judgment) and item 6 (overidentification). Although the latter correlation was not between items from the same subscale, it appears reasonable given that self‐judgment and overidentification did not form separate factors in our data. The full output of all investigated BP models can be found in the OSF (Table S1).

### 4.2 Within‐person models

Table 4 presents the model fits of the competing models at the WP level. Overall, the WP models had similar fits to the BP models. As at the BP level, only Model 7 met criterion 1, but the correlation between self‐judgment and isolation (*r* = 1.01) also questioned the validity of this model at the WP level. However, also the five‐factor model had a non‐positive definite covariance matrix due to a small negative eigenvalue. Since the loadings and variances did not show any anomalies, we suspect that this might be caused by the factor correlations. As at the BP level, the correlations were high within the factors of CS (*r* = 0.50 to 0.87) and within the factors of UCS (*r* = 0.81) but not between them (cf. Table 3, above the diagonal). In contrast to the BP level, there were no substantial negative correlations at the WP level, and self‐kindness did correlate positively in medium strength with both factors of UCS (*r* = 0.46 to *r* = 0.52). The high correlation between mindfulness and common humanity seemed to cause the issue with the five‐factor model since merging those factors resolved the issue. The resulting four‐factor model seemed to fit the data adequately and was thus preferred over the six‐ and five‐factor solution. Despite these differences in the BP level, the correlation pattern at the WP level also clearly suggests two general factors instead of one.

**TABLE 4:** Model fits of the competing models at the WP level.

No.	Model type	CFI	RMSEA	df	*χ* ^*2*^	*p*	Note
1	One‐factor model	0.746	0.115	54	1898.356	<0.001	–
2	Two‐correlated factor model	0.895	0.075	53	856.800	<0.001	–
2^a^	Two‐correlated factor model with one residual correlation	0.921	0.065	52	641.040	<0.001	Exploratory model
3	One higher‐order factor model	0.834	0.093	54	1239.116	<0.001	OI negative variance
4	Two‐correlated higher‐order factor model	0.899	0.073	53	813.639	<0.001	SK and OI negative variance
5	One bifactor model	0.858	0.091	48	1107.424	<0.001	OI negative variance
6	Two‐correlated bifactor model	0.942	0.059	47	512.505	<0.001	SK and OI negative variance
7	Six‐correlated factor model	0.937	0.063	45	502.684	<0.001	SJ and OI correlate >1.0
7^a^	Five‐correlated factor model	0.932	0.061	51	538.164	<0.001	Negative eigenvalue
7^a^	Four‐correlated factor model	0.921	0.063	56	607.760	<0.001	Exploratory model

*Note*: Robust values for the model fit are displayed.

Abbreviations: OI, overidentification; SJ, self‐judgment; SK, self‐kindness.

^a^ Modified version of the preregistered model.

Consistently, models with two general factors always fit better than the corresponding model with one general factor. None of the models with one general factor demonstrated an acceptable fit, whereas the two‐factor model (Model 2) showed a close fit, and the two bifactor models (Model 6) showed a good fit but two specific factors had negative or non‐significant variances.^7^ The misfit in Model 2 was attributable to the measurement model of CS (*χ* ^*2*^ = 388.053, *p* < 0.001, CFI = 0.836, RMSEA = 0.116) and not to the model of UCS (*χ* ^*2*^ = 97.654, *p* < 0.001, CFI = 0.975, RMSEA = 0.072). Including the first recommended residual correlation for the mindfulness, items was sufficient to reach an acceptable fit. The full output of all investigated WP models can be found in the OSF (Table S2).

### 4.3 Comparison of between‐ and within‐person level

Overall, the configural structure of self‐compassion appeared to be similar across levels. At the WP and the BP levels, modified versions of Model 2 and Model 7 met the minimal criterion. Yet in both cases, the models can only be considered partially configural invariant. For Model 2, the BP model contains two additional residual correlations for an acceptable fit according to the CFI. In contrast, the RMSEA already indicated a good fit at the BP level without any correlated residuals. The “disagreement” of both fit indices could be caused by sampling variation, which has been shown to impact the CFI more strongly than the RMSEA (Lai & Green, 2016). This seems plausible given that the sample size at the BP level was small, and both fit indices disagreed less strongly at the WP level where the sample size was way larger. At the same time, the high magnitude of all residual correlations at the BP level suggests that the CFI was low due to the unmodeled variance in the items. Thus, the items of the same subscale may share more unique variance at the BP level than at the WP level. For Model 7, we had to merge two additional factors in the WP model to achieve a satisfying solution. That is, although the correlation between mindfulness and common humanity was only slightly higher at the WP level (*r* = 0.87) than at the BP level (*r* = 0.83), it seemed to cause a non‐positive definite covariance matrix in the five‐factor model at the WP level which is why those factors were combined at this level. Thus, also those results suggest that there might be weaker evidence for subscale factors at the WP level compared to the BP level.

As the final model, we chose Model 2 over Model 7 in line with our preregistration (cf. criterion 2). We preferred a model with at least one general factor over (e.g., Model 2) over the six‐factor model (Model 7) because six subscale scores are rarely used in studies, but it is instead common to compute one or two total scores (Muris & Otgaar, 2020). Nevertheless, we can only follow our preregistration in a conceptual and not in a strict sense because both models had to be modified to meet the minimal criterion.

**FIGURE 2:**
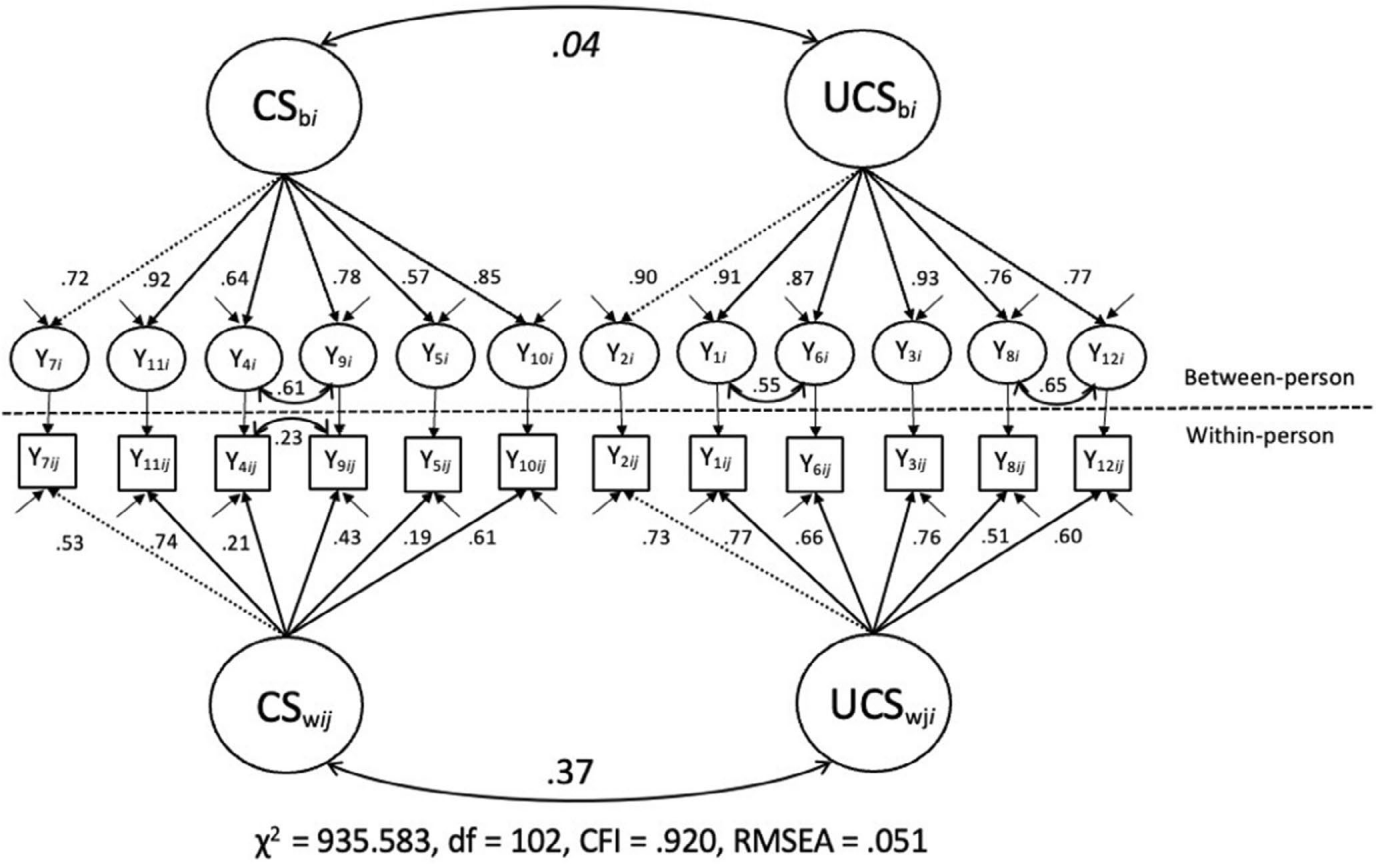
Modified version of Model 2 at the BP and the WP levels. Standardized parameters and robust model values are displayed. Dashed lines represent loadings that were fixed to 1. Non‐significant parameters (*p* < 0.05) are in italics. Estimates of residual variances are not displayed. CS = compassionate self‐responding; UCS = uncompassionate self‐responding.

For a comparison of the parameters at both levels, we specified the final model simultaneously at both levels (cf. Figure 2). As expected in MCFA, the standardized loadings are higher at the BP level than at the WP level due to smaller standard errors since estimates of means are more reliable than estimates of variability (Biesanz et al., 2003). Importantly, the relationship between CS and UCS differed descriptively across both levels: At the BP level, CS and UCS were not substantially related (*r* = 0.04, *p* = 0.696), whereas they were moderately correlated at the WP level (*r* = 0.37, *p* < 0.001).

### 4.4 Robustness checks

Self‐compassion describes, by definition, how one relates to oneself in *difficult times*, which is usually reflected in the item text of the SCS (e.g., “When I'm feeling down…”; Neff, 2003b). Yet, the adapted version of the items that we used in the present study did not entail a description of the situation, leaving it unclear whether participants' responses referred to a difficult situation (and thus applied to the definition of self‐compassion). To investigate how this uncertainty might have impacted the results, we repeated all analyses in a subset only containing measurement occasions when participants indicated that they had just experienced a stressful event. We reasoned that in this “stress subset,” the items should have been applied to the situation by the definition of the construct. In the stress subset, the results were overall comparable, with the most notable difference being the correlations between the CS and UCS items: While the overall correlation pattern still indicated the existence of two factors, the correlations between CS and UCS were descriptively stronger than in the entire dataset. The latent factors were moderately correlated at the BP level (*r* = 0.279, *p* = 0.026), and strongly correlated at the WP level (*r* = 0.510, *p* < 0.001). Moreover, we conducted additional robustness checks such as fixing the negative variance in the models to 0 or only including participants who completed at least 90% of the reports. In both cases, the results did not change substantially. The full results of the robustness checks are reported in the OSF.

### 4.5 Exploratory investigation of the heterogeneity in the within‐person relationship

In addition to the average WP structure obtained through MCFA, we conducted exploratory analyses to estimate the heterogeneity of the WP association. To this end, we used multilevel regression models at the manifest level in which random slopes can be easily implemented (as opposed to SEM models). To overcome the asymmetry of regression analyses, we person‐mean standardized the composite scores of CS and UCS (Schuurman et al., 2016). Due to WP standardization, the individual slopes^8^ conceptually represent the WP correlation between the CS and UCS.

**FIGURE 3:**
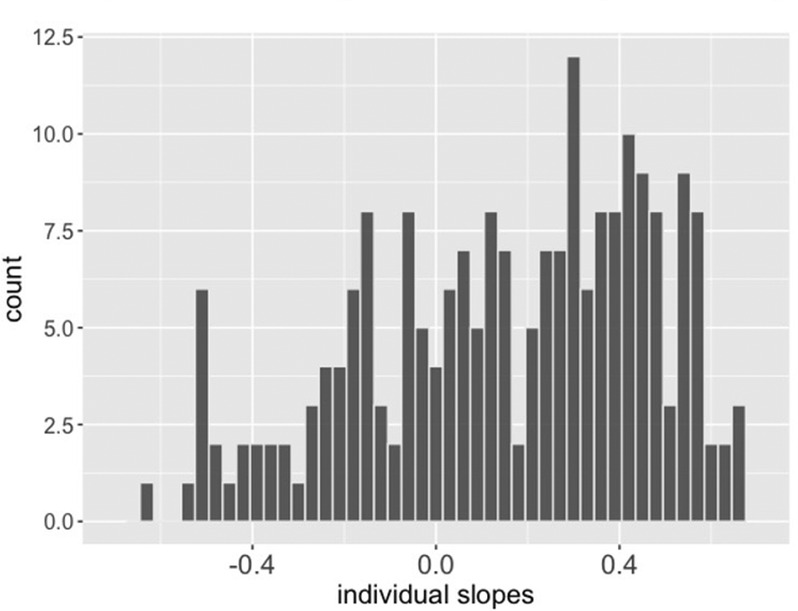
Model implied individual slopes between the composite scores of the CS and UCS. The values were within‐person standardized before the analyses the slopes hence represent conceptually the individual correlation between composite scores of CS and UCS. *N* = 211 participants.

As illustrated in Figure 3, the individual slopes showed considerable variance (*SD* = 0.31, *Range* [−0.62; 0.70]). Comparing the mean of the slopes (*β* = 0.16) with the average latent correlation (*r* = *0*.37) gives a rough estimation of how strongly the manifest slopes are attenuated by measurement error. Against this background, it should be noted that the variance and not the absolute values of the slopes were of main interest in this exploratory investigation.

To gain first insights into the possible meaning of interindividual differences in the intraindividual relationship, we correlated the individual slopes with the individual mean and standard deviation of the composite scores of CS and UCS (cf. Table S12 in the OSF). The WP relationship was stronger for participants with a higher mean on CS (*r* = 0.42, *p* < 0.001), and weaker for participants with a higher mean on UCS (*r* = −0.24, *p* < 0.001). Moreover, the WP relationship was stronger for participants with greater intraindividual variability on UCS (*r* = 0.21, *p* = 0.002). This finding likely demonstrates the floor effect of the UCS items that restricted the WP variance. That is, CS and UCS cannot vary together within an individual (i.e., correlate) if the person does not show sufficient variation on the UCS items. Thus, the WP correlation between CS and UCS may be attenuated due to the low WP variance on the UCS items. Supporting this interpretation, the intraindividual variability of the CS items—which was on average larger than for the UCS items—was not significantly related to the WP relationship (*r* = 0.05, *p* = 0.508). To check for further method effects, we correlated the absolute values of the slopes with the number of measurement occasions per participant but did not find evidence for a substantial relationship (*r* = 0.08, *p* = 0.220).

## 5 DISCUSSION

In this paper, we empirically illustrate the benefits of distinguishing levels of analysis when defining psychological constructs, using self‐compassion as an example. The factor structure of self‐compassion as a trait (i.e., dominated by BP variance) has been debated and frequently investigated, but we point out that the theory of the construct is actually concerned with relationships at the WP level. To examine the implications derived from this assumption, we measured self‐compassion as a state in daily life and separated the WP structure from the BP structure. To further illustrate the insights that can be gained by the WP perspective, interindividual differences in the intraindividual relationship between the components of self‐compassion were investigated in exploratory analyses.

### 5.1 Summary of results and implications for the debate around self‐compassion

Our results suggest largely configural but not a structural correspondence of the factor structure of self‐compassion across the WP and BP levels. Two models provided an acceptable fit after similar modifications: First, the two‐factor model (Model 2) required one or three residual correlations for items of the same subscale at the WP and BP levels, respectively; second, the initial six‐factor model (Model 7) required reducing the number of factors to four or five factors at the WP and BP levels, respectively. As the final model, we chose the modified two‐factor model, in line with the preregistered decision criterion that reflects theoretical and practical considerations. The six‐factor model provides an adequate fit in most studies, and in some, it is also the only model that does so (e.g., Wiliams et al., 2014; Hupfeld & Ruffieux, 2011). Yet, six subscale scores as implied by this model are rarely used in studies; instead, it is common to compute a composite score across subscales (Muris & Otgaar, 2020). Consequently, we preferred models with at least one general factor that supports the computation of composite scores.

One central question that is currently being debated is how composite scores for self‐compassion scales should be computed—is one or are two total scores more appropriate to describe the self‐compassion of an individual? If measured as a state in daily life, the factor structure at the BP and WP levels clearly implies the computation of two total scores (CS and UCS). The two‐factor solution always fitted better than the corresponding one‐factor solution at both levels of analysis.

Yet, the strength of the relationships between the two factors depended on the level of analysis: CS and UCS were largely independent at the BP level (*r* = 0.04, *p* = 0.695), but moderately correlated at the WP level (*r* = 0.37, *p* < 0.001). In other words, the results suggest that across situations, people can have the tendency for compassionate *and* uncompassionate self‐responding, but within a given situation, they rather treat themselves in either a compassionate *or* uncompassionate manner. For this interpretation, it is important to remember that items of UCS were reverse scored. That is, the results suggest that increased CS is associated with decreased USC responding at the moment and vice versa. Note that the absolute values of the correlation were likely attenuated at both levels of analysis due to the study design. Participants were asked at random to respond to the adapted self‐compassion items, leaving it unclear how applicable the items were to their current situation. Thus, the analyses were repeated in a subset of observations in which participants had just experienced a stressful event. In this stress‐subset, the correlations between CS and UCS were higher, likely due to different responses to the UCS items. The UCS items had more variance in the stress‐subset because participants used the most extreme option less consistently, suggesting the items may have been more applicable to the situation (Kritzler et al., 2023) in the face of a stressful event. However, this does not affect our conclusion about structural differences between levels since also in the stress‐subset, the correlations at the WP level (*r* = 0.510, *p* < 0.001) were descriptively higher than at the BP level (*r* = 0.279, *p* = 0.026). The finding that the relationships between factors are stronger at the WP level than at the BP level aligns with past studies on effect (e.g., Bleidorn & Peters, 2011; Merz & Roesch, 2011) and personality states (Ringwald et al., 2022), and empirically underlines the need to distinguish between the WP and BP levels.

The results from exploratory analyses point toward another reason why it may be useful to keep CS and UCS as distinct scores. We found that the WP relationship between CS and UCS differed greatly between participants, suggesting that compassionate and uncompassionate self‐responding seem to function in a bipolar manner for some individuals but rather independently for others. These findings of state self‐compassion mirror those of trait self‐compassion from Ferrari et al. (2022) who found great heterogeneity in the correlation of WP changes of self‐kindness (component of CS) and WP changes in self‐judgment (a component of UCS) across adolescents. Such interindividual differences in the WP relationship could not be detected if a total score was used. But what do interindividual differences in the WP relationship between CS and UCS tell us?

A possible suggestion on the meaning of interindividual differences in the WP relationship between CS and UCS may be found when turning toward another result of our exploratory analyses. We found that the composite scores of CS and UCS were moderately related to the strength of the WP relationship, which is interesting considering the current discussions around the conceptualization of self‐compassion. It was argued that only CS reflects the protective nature of self‐compassion, whereas UCS reflects (vulnerability for) psychopathology (Muris, 2016; Muris et al., 2019; Muris & Petrocchi, 2017). Applying this conceptualization, the results of our study suggest that increased CS is associated with decreased UCS—as implied by the initial theory (Neff, 2003a)—but particularly for individuals with high dispositional levels of self‐compassion (i.e., high composite scores of CS). In contrast, our findings indicate that individuals with high dispositional vulnerability (i.e., high composite scores of UCS) display a weaker link between compassionate and uncompassionate responding. A weak or even negative link between CS and UCS may indicate that people *try* to treat themselves compassionately (e.g., by reminding themselves that all humans are flawed) but still experience the presence of negative thoughts and feelings (e.g., feel that most people are happier than themselves). This is supported by the item text: The positive items appear to reflect adaptive attempts (all but one positive item begins with: “I am trying…”), whereas the negative items reflect current maladaptive behaviors (e.g., “I am obsessed…” or “I am fixating…”).^9^ As such, the WP correlation may indicate how successful an individual's attempts are on average to treat themselves compassionately.

The interpretation that the WP relationship may indicate how effective an individual's attempts are to treat themselves compassionately may offer a fresh take on the debated role of UCS for self‐compassion. According to Neff (2020, 2022), interventions of self‐compassion particularly focus on enhancing CS instead of decreasing UCS because “it is more effective to teach clients what to do (be kinder, remember common humanity, and be mindful) than what not to do (be less judgemental, feel less isolated, stop overidentifying)” (Neff, 2020, p. 1904). In other words, interventions of self‐compassion are designed to decrease UCS by increasing CS. Against this background, it may be most useful to define self‐compassion only in terms of CS as it entails concrete strategies that people can learn in interventions, and use the maladaptive thoughts and behaviors covered by UCS as outcomes to evaluate the effectiveness of CS.

To summarize, although this study finds that the factor structure of self‐compassion is comparable across the BP and WP levels, it also shows the benefit of distinguishing both levels of analysis. The difference in the relationship between CS and UCS across levels of analysis demonstrates that empirical results based on cross‐sectional data on self‐compassion measured as a trait provide limited insights on the WP relationship, which lies at the core of the theory as we argue. While we only obtained estimates for the average WP relationship in our preregistered analyses, exploratory analyses indicate substantial variation in the WP relationship across individuals, which may inspire theoretical refinements. For example, for whom and when does increased CS lead to decreased UCS? Answers to such questions would provide useful knowledge for interventions. Yet, such questions can only be examined from a WP perspective. Thus, perhaps the most important implication of this study for the debate around self‐compassion would be to shift away from cross‐sectionally studying self‐compassion as a trait toward understanding the construct from a WP perspective.

### 5.2 More states, fewer traits?

Our results demonstrate once more that the meaning of variables can differ across levels of analysis. This leads to the question of whether conceptualizing constructs as states rather than traits would be more beneficial when WP variation plays a significant role in the theory at hand. Until now, it seems that constructs are routinely conceptualized as traits in personality psychology even if they are like self‐compassion, for example, thought of as an “adaptive process” (Neff, 2003b, p. 235). But what would it mean for a construct to be explicitly conceptualized as a state?

First, conceptualizing constructs as states means that situational or transient factors represent a meaningful source of variation (Horstmann et al., 2021). Interindividual differences in the perception and reaction to these situational factors (i.e., person–situation interactions) also represent an important aspect of the nomological net of the state of interest. For example, for self‐compassion, the momentary source of suffering appears to be a particularly relevant situational factor. According to the trait perspective on self‐compassion, self‐compassionate individuals treat themselves in a compassionate manner regardless of the source of suffering (Neff, 2003a). However, this proposed consistency in the reaction to challenging experiences has never been empirically investigated. Instead, it appears likely that individuals vary in how they respond to themselves (i.e., compassionately vs. uncompassionately) depending on the momentary cause of their suffering. There may be shared variation across people, such that perhaps most people have an easier time being understanding towards the self if the suffering is caused by something external (vs. caused by themselves), as well as idiosyncratic variation, such as that a person may only be particularly harsh to themselves when they are confronted with insecurities at work. Such a shift in the conceptualization from trait to state could have meaningful implications for interventions. For example, if people learn about self‐compassion as a response to difficult experiences that vary across situations, they can be encouraged to find out for themselves in which situations they particularly show vs. lack self‐compassion. As this example demonstrates, conceptualizing constructs as states offers new ways of theorizing that could be particularly beneficial for interventions.

Second, conceptualizing constructs as states means that study designs with repeated assessments should be favored over cross‐sectional designs. Only data with repeated observations allow examining the nomological net of a state in which the WP and BP levels are both considered but kept distinct. Moreover, WP variation may also be useful to derive estimates at the BP level (often called “WP indices”) that allow a richer or more accurate description of interindividual differences in the construct as they unfold in daily life (see Dejonckheere et al., 2019 for an overview in affective science). For example, while the results on the heterogeneity in the WP relationship between CS and UCS call for further research on the robustness and meaning of those interindividual differences, the field of affective sciences suggests that such endeavors can be worthwhile. The WP relationship between positive and negative affect (i.e., affective bipolarity) has been shown to reliably predict well‐being above mean levels (Dejonckheere et al., 2019) and to serve as a specific marker of depression (Dejonckheere et al., 2018). Affective bipolarity was suggested to reflect the degree of differentiation with which people process emotional information such that it becomes stronger when limited cognitive resources (e.g., in times of stress) require simplified processing (Davis et al., 2004; Zautra et al., 1997, 2000, 2002). Thus, (some) WP indices may not only improve the prediction of important outcomes but can also lead to theoretical refinements.

Lastly, conceptualizing constructs as states means that the development and validation of methods to estimate WP variation are essential. While instruments to measure traits were developed and validated to distinguish between individuals, instruments to measure states need to validly capture variation within individuals across occasions. For example, the state self‐compassion scale was developed to detect WP changes in state self‐compassion after experimental manipulations (Neff et al., 2021). Accordingly, results suggesting that the scale scores reliably capture change after a short self‐compassionate mindset induction serve as valid evidence for the scale scores. However, such evidence tells us little about whether the measure is also able to detect changes in self‐compassion in daily life that are likely caused by a broad range of factors and unfold in various situations. That is, state measures that are validated for experimental settings are not automatically suited for ESM designs and vice versa. Instead, as for trait measures, the intended use of the state measure should guide the development process and validity strategy (Horstmann & Ziegler, 2020; Ziegler, 2014). Importantly, the development of accurate measures not only represents the foundation for the empirical study of WP variation but can also enhance the accuracy of the underlying theory (Scheel et al., 2021).

### 5.3 Limitations

The results of our study may serve as a starting point for further endeavors that examine self‐compassion (and other constructs) from a WP perspective. In this context, the following limitations should be kept in mind. First, we adapted the items of the SCS for the context of an ESM study because no measure of state self‐compassion exists that has been validated for daily life research. While the item adaptation aimed to change the wording of the original items from the SCS as little as possible, the UCS items appeared to be suboptimal for studying self‐compassion as a state in daily life since most individuals disagreed with the items most of the time. This has led to little variance at both levels of analysis and likely attenuated the correlations. Second, in our study design, participants were not explicitly asked to respond to the items regarding a difficult situation they experienced or a challenging thought, leaving it unclear how the participants responded to the items. We tried to account for this post‐hoc by repeating the analyses in a subset of the measurement occasions when participants indicated that they had just experienced a stressful event. Nevertheless, stressful events represent only specific types of situations, whereas it is an essential aim for research on state self‐compassion to capture the full range of situations that may elicit (un)compassionate self‐responding. Third, some results should be interpreted as exploratory as post hoc modifications of the models (e.g., including correlated residuals) were required. Nevertheless, we believe that the finding of configural but not structural correspondence between the average WP and BP structure of self‐compassion is robust.

Fourth, we only obtained estimates about the *average* WP structure. That is, the same WP structure is assumed for all individuals, which is a false assumption given the variation of WP association between CS and UCS that we found in exploratory analyses. Based on studies that applied a fully idiographic approach (e.g., Beck & Jackson, 2020; Brose et al., 2015; Hamaker et al., 2005), it is likely that not only the WP associations between CS and UCS vary across individuals but also the loadings and the number of factors. Lastly, we based the conclusion on the structural correspondence only on a descriptive comparison. Since the BP and WP levels differed strongly in their sample size, we considered an inferential test of the equivalence of both levels unreasonable. The absence of an “objective” criterion and the application of our subjective reasoning regarding the difference between the structure at both levels means that readers may conclude the current study results differently.

### 5.4 Conclusion

This study demonstrates that the structure of self‐compassion meaningfully differs across the WP and BP levels. As such, it underscores the importance of aligning the level of analysis between methods and theories in psychological research to increase the utility of empirical research to test and ultimately improve psychological theories. To increase such an alignment, psychological theories should clearly state at which level of analysis a given phenomenon is expected to take place. The level of analysis of interest should then guide the conceptualization and assessment of the constructs that lie at the core of the theory. This may require further development in the current operating procedure in personality psychology to conceptualize and measure (almost) all constructs as traits. Instead, it may be beneficial to conceptualize constructs such as self‐compassion as states in further research.

## AUTHOR CONTRIBUTIONS

A.B.: Conceptualization, Data curation, Methodology, Formal Analysis, Writing – Original Draft. C.E.: Data curation, Supervision. C.F.A.H.: Data curation. M.S.A.: Funding acquisition, Methodology, Writing – Review & Editing, Supervision. K.T.H.: Conceptualization, Methodology, Writing – Review & Editing, Supervision.

## CONFLICT OF INTEREST STATEMENT

The authors have no conflict of interest to disclose.

## ETHICS STATEMENT

All procedures in this study involving human participants adhered to the ethical standards of the German Research Foundation and were in accordance with the Helsinki Declaration of 1975, as revised in 2013. Given the study's reliance on anonymized data from an adult general population sample without involving vulnerable groups or inducing significant psychological stress, physical pain, or exceptional risks, an ethics committee review was deemed unnecessary. This exemption aligns with the guidelines provided by the German Research Foundation and the ethics committee of the University of Potsdam. For further details, see https://www.dfg.de/en/research_funding/faq/faq_humanities_social_science/index.html

## Data Availability

The analyses of this study were preregistered (https://osf.io/2p7sq/). The study materials, data, and analysis scripts used for this article can be accessed at https://osf.io/fbcwp.

## Endnotes

^1^
The different “components” of self‐compassion could be also referred to as facets. However, in the self‐compassion literature, the term “components” is most commonly used.

^2^
These sample characteristics refer to *n* = 211 because for two participants the ESM data could not be matched with the trait data that contained the demographic information.

^3^
To check the robustness of our decision to focus on the CFI and RMSEA, we also report the AIC and BIC that penalize model complexity in the OSF in Table S1 and S2. The AIC and BIC suggest the same conclusion than the CFI and the RMSEA.

^4^
To address common issues of models with a general factor (i.e., higher‐order and bifactor models) such that at least one specific factor has a negative or non‐significant variance estimate, we considered the S‐1 model (Eid et al., 2017), which has not been tested before for self‐compassion. Yet, since specifying the higher‐order and bifactor models as S‐1 models did not help with the issues of the models in our study, the rational and precise specification of the models is only presented in the OSF. A brief explanation why we did not use exploratory structural equation modeling (ESEM; Asparouhov & Muthén, 2009) despite suggestions that this is the most appropriate modeling approach for self‐compassion (Neff et al., 2019) can also be found in the OSF.

^5^
It may be that two factors for CS and UCS emerged due to method effects of the item wording. A study using an MTMM approach indeed reported that the CS and UCS items formed separate factors, suggesting method effects (Montero‐Marin et al., 2018). However, since the study did not assess other relevant constructs (e.g., neuroticism and psychopathology; Muris, 2016; Pfattheicher et al., 2017), it cannot be ruled out that the separate factors for the items of CS and UCS represent substantive constructs instead of method factors. To date, it is commonly assumed that the two factors reflect substantive constructs in the literature on self‐compassion (e.g., Muris & Otgaar, 2020; Neff, 2020).

^6^
One specific factor still had a negative variance estimate if the model was tested as S‐1 model (Eid et al., 2017). See the OSF for the full results.

^7^
Again, Model 6 specified as S‐1 model did not fulfill the minimal criterion due to the negative variance of one specific factor.

^8^
Two participants were excluded from the analyses because they did not vary on any of the CS items (i.e., the individual standard deviation was 0). Thus, the individual slopes were only calculated for 211 participants.

^9^
Importantly, this difference in the formulation between the items of the positive and negative components stems from the original items of the SCS and is not the result of the adaption of the items undertaken in the current study. Thus, it may be that the difference in the wording of the positive and negative items leads to a lower correlation between both factors more generally. This may also explain why such a high correlation between the positive and negative factors for the newly developed state self‐compassion scale (Neff et al., 2021) was found because for this scale, the ‘trying’‐part of the positive items was removed.
